# Generation and Characterization of Single-Domain Antibodies Against the D-Antigen of Sabin Poliovirus Types 2 and 3

**DOI:** 10.3390/v18101147

**Published:** 2026-10-16

**Authors:** Maya Ermakova, Sergey Ivanov, Olga Shmeleva, Nadezhda Borisenko, Anna Zyrina, Igor Levin, Stepan Chumakov, Anna Shishova, Yury Ivin, Anastasia Piniaeva, Alexandra Siniugina, Aydar Ishmukhametov

**Affiliations:** 1 Chumakov Federal Scientific Center for Research and Development of Immune-and-Biological Products, Russian Academy of Sciences (Institute of Poliomyelitis), 108819 Moscow, Russia; ivanov_sv@chumakovs.su (S.I.);; 2 Shemyakin-Ovchinnikov Institute of Bioorganic Chemistry, Russian Academy of Sciences (IBCh RAS), 117997 Moscow, Russia; 3 Institute for Translational Medicine and Biotechnology, First Moscow State Medical University (Sechenov University), 119991 Moscow, Russia

**Keywords:** poliovirus, Sabin, VHH, phage display, D-antigen, ELISA test, neutralization

## Abstract

A panel of monoclonal nanobodies (VHH) specific to poliovirus Sabin strains types 2 and 3 has been generated and characterized. For poliovirus Sabin type 2, a panel of four serotype-specific single-domain antibodies was obtained. Notably, two of these antibodies, S2-C5 and S2-D5, were able to discriminate between D- and H-antigens. Three nanobodies specifically neutralized poliovirus Sabin type 2 and exhibited no neutralizing activity against the homologous wild-type MEF-1 strain or other poliovirus serotypes. Among the four nanobodies selected against Sabin type 3, S3-A2 and S3-G1 demonstrated high serotype specificity in ELISAs. When used as a capture antibody in the ELISA, S3-G1 showed preferential binding to D-antigen over H-antigen. However, the type 3 nanobodies did not display virus-neutralizing activity. The obtained antibodies represent promising tools for improving existing immunoassays based on polyclonal antibodies by increasing D-antigen specificity and may facilitate the development of next-generation ELISA systems for the quality control of inactivated polio vaccines.

## 1. Introduction

Vaccination remains the primary and most effective method of poliovirus infection prevention [1]. Due to mass immunization, significant progress has been achieved by 2026, according to the Global Polio Eradication Initiative (GPEI). Wild poliovirus types 2 and 3 have been officially declared globally eradicated [2]. Cases caused by wild poliovirus type 1 are now reported only in a limited number of endemic countries [3]. However, in addition to wild strains, circulating vaccine-derived polioviruses (cVDPVs), particularly type 2 (cVDPV2), as well as cases of vaccine-associated paralytic poliomyelitis (VAPP), pose a threat following the administration of oral (live) poliovirus vaccine (OPV) [4,5]. Furthermore, there is a risk of immunodeficiency-associated vaccine-derived polioviruses (iVDPVs) in individuals with B-cell immunodeficiency [6]. For this reason, preference is given to the use of the inactivated polio vaccine (IPV), which cannot cause VAPP or generate cVDPVs and iVDPVs. Accordingly, the GPEI has developed a strategy that entails the gradual phase-out of OPV from routine immunization and a transition to IPV, to be aligned with the complete certification of wild poliovirus eradication and the cessation of circulation of all VDPVs [7].

Currently, trivalent IPV based on Salk strains (Mahoney type 1, MEF-1 type 2, and Saukett type 3) and IPV based on attenuated Sabin strains (LSc 2ab type 1, P712 Ch 2ab type 2, and Leon 12a1b type 3) are in use. The key component of IPV is the D-antigen–a native conformational virion that retains an intact capsid structure and viral RNA. This component induces the production of virus-neutralizing antibodies and confers the specific activity of the vaccine [8]. The immunogenicity of the D-antigen is determined by the presence of conformational epitopes on the surface of the viral particle, which is composed of 60 protomeric units of four capsid proteins: VP1, VP2, VP3, and VP4 [9]. The spatial arrangement of these proteins defines the serotype specificity and immunogenicity of poliovirus [10]. In addition to the mature form (D-antigen), polioviruses obtained from cell cultures may also be present as immature empty capsids lacking RNA. Such particles, termed C-antigen, differ in antigenic composition and do not induce an immune response [11]. This heterogeneous group includes immature particles formed during virus replication and empty capsids generated by the release of viral RNA at late stages of infection [12]. Although the C-form and the heat-induced H-form are not identical in terms of their origin, they share related non-native capsid conformations and antigenic properties. During IPV production, the native RNA-containing D-antigen can undergo a conformational transition to an H-like, non-native antigenic state under stress conditions, such as heating, including treatment at up to 56 °C, or formalin inactivation [13,14]. In this study, we use the term H-antigen specifically because the particles were generated experimentally by heat treatment of the native D-antigen.

Accurate quantification of the D-antigen is critically important for the quality control and efficacy of vaccine batches. Various immunochemical test systems calibrated against WHO international standards have been developed for this purpose [14]. Routine standardization and the high batch-to-batch variability inherent to polyclonal sera can lead to substantial errors in the assessment of target antigen concentration and may affect the immunogenicity of vaccine lots. The development of test systems based on monoclonal antibodies overcomes these limitations, as the resulting recombinant clones exhibit high specificity for a single epitope and, owing to their genetic identity, ensure greater assay reproducibility [15].

Understanding the structural organization of neutralizing epitopes is crucial for the development of such specific reagents. In the fundamental studies by Minor and colleagues, three major antigenic sites (sites 1, 2, and 3) recognized by virus-neutralizing monoclonal antibodies have been identified on the surface of the poliovirus capsid [16,17] (Supplementary Figure S3). Antigenic site 1 is located in VP1 (residues 89–100) and is immunodominant for serotypes 2 and 3, whereas it is immunorecessive for serotype 1. Site 2 is a complex, discontinuous epitope combining residues 220–222 of VP1 (subsite 2a) with residues 164–172 and 270 of VP2 (subsite 2b). Both subsites are active in serotypes 1 and 2, while in serotype 3 only subsite 2b is distinctly detected [17,18,19]. Site 3 is also complex and comprises subsite 3a in VP1 (residues 286–290) and subsite 3b in VP3 (residues 58–59 and others). Both subsites 3a and 3b are present in serotypes 2 and 3, whereas only subsite 3b has been found in serotype 1 [17,20] (Supplementary Figure S3). Such inter-serotype differences in epitope composition underscore the need for antibodies specific to the conformational epitopes of the D-antigen of each individual serotype, which is particularly relevant for the development of test systems for the quality control of IPVs produced from both wild-type and attenuated strains.

In this study, monoclonal antibodies were generated using phage display technology, which relies on the expression of target proteins on the surface of filamentous M13 bacteriophages as fusions with the p3 coat protein [21]. Using this method, single-chain VHH antibodies (nanobodies) from camelids, consisting of the variable domain of the heavy chain, were obtained and characterized [22]. A unique feature of VHHs is their hypervariable CDR3 domain, which is typically the longest and forms a protruding loop. This structure enables nanobodies to bind otherwise inaccessible epitopes, which is of particular interest given the structural diversity of conformational viral epitopes [23]. VHH antibodies may offer advantages over conventional monoclonal antibodies because of their small size, high stability, and ability to access sterically restricted epitopes. In addition, they can be readily engineered and combined into multivalent or multispecific constructs. The literature reports the generation and characterization of nanobodies against poliovirus type 1, including their structures in complex with the virus and their neutralizing properties [24,25]. However, these studies were limited to poliovirus type 1, and nanobodies against poliovirus serotypes 2 and 3 have not been described to date.

Previously, a panel of highly specific nanobodies against poliovirus Sabin type 1 was generated at the Chumakov Federal Scientific Center (Institute of Poliomyelitis) [26]. The present study is a continuation of that work. It describes, for the first time, the generation and functional characterization of a panel of nanobodies specific to the D-antigens of poliovirus Sabin strains types 2 and 3. The obtained antibodies were tested by ELISA for their ability to recognize the D-antigen of the corresponding serotype, as well as in a virus neutralization assay.

## 2. Materials and Methods

### 2.1. Production of Purified Poliovirus and Animal Immunization

Sabin poliovirus strains P712 Ch 2ab (type 2) and Leon 12a1b (type 3) were propagated in Vero cells using the standard production process, including cultivation in roller bottles and bioreactors, clarification of virus-containing fluid, sterile filtration, and concentration by tangential-flow ultrafiltration [27]. Virus concentrates with infectious titers of at least 10^10^ TCID_50_/mL were purified by cesium chloride density-gradient ultracentrifugation. Gradient fractions corresponding to D-antigen were collected and desalted by gel filtration. Purified viral preparations were inactivated with 0.03% formaldehyde in Medium 199 for 13 days at 37 °C as previously described [28].

Clinically healthy adult alpacas (*Vicugna pacos*) were housed under standard husbandry conditions with unrestricted access to food and water and routine veterinary supervision. Animals were immunized intramuscularly with purified inactivated D-antigen (≥5000 DU) emulsified in Freund’s adjuvant (Thermo Fisher, Waltham, MA, USA) according to a three-dose schedule consisting of one primary immunization followed by two booster injections at three-week intervals. The D-antigen content was quantified using the assay described before, calibrated against the WHO international Standard for Inactivated poliomyelitis Vaccine 17/160 [28]. Blood samples were collected before immunization and after completion of the immunization course for assessment of the humoral immune response and subsequent isolation of peripheral blood mononuclear cells.

### 2.2. Construction, Selection and Screening of VHH Phage Libraries

Peripheral blood mononuclear cells (PBMCs) were isolated from immunized alpacas and used for total RNA extraction. First-strand cDNA was synthesized using MuMLV reverse transcriptase (Evrogen, Moscow, Russia) and the primer specific for camelid heavy-chain immunoglobulin variable domains (CH2-IgG–sp_rev: 5′-GGTACGTGCTGTTGAACTGTTCC-3′). The resulting cDNA was amplified by PCR using primers containing XbaI and BamHI restriction sites to enable subsequent cloning (F1X: 5′-GATCTCTAGAATGCAGKTGCAGCTCGTGGAGTCNGGNGG-3′; R1B:5′-GATCGGATCCGGTTGTGGTTTTGGTGTCTTGGG-3′; R2B:5′-GATCGATCCGGGGGGTCTTCGCTGTGGTGCG-3′). PCR products were digested with *XbaI* and *BamHI*, ligated into the pHEN2-XB phagemid vector, and transformed into *E. coli* TG1 (Lucigen, Middleton, WI, USA) cells by electroporation. Library size and transformation efficiency were estimated by colony counting, the presence of VHH inserts was verified by colony PCR.

Recombinant phage particles were generated by rescue with M13KO7 (NEB, Ipswich, MA, USA) helper phage, precipitated with PEG/NaCl, resuspended in PBS containing glycerol, and titrated by serial dilution.

The general selection procedure can be described as follows. D-antigens of Sabin poliovirus types 2 and 3 (50 DU/mL) were immobilized separately on polystyrene tubes. A phage display library of VHH antibodies was added to the immobilized antigen in blocking solution, 1% skim milk solution (AppliChem, Darmstadt, Germany) in PBS-T (ServiceBio, Wuhan, China), and incubated for 1.5 h. After incubation, unbound phages were removed by washing the tubes three times with PBS-T buffer. After each selection round, bound phages were eluted under alkaline conditions, by the addition of 100 mM triethylamine (pH 11.0, Ruschem, Russia), amplified in *E. coli* TG1 cells, and subjected to the subsequent round of enrichment.

Enriched phage libraries were screened by phage ELISA using D-antigens of all three Sabin poliovirus serotypes. Positive clones were identified based on serotype-specific binding and signal-to-background ratios exceeding 30-fold for Sabin type 2 and 35-fold for Sabin type 3. VHH inserts of selected clones were sequenced by the Sanger method (ABI3500, Applied Biosystems, Foster City, CA, USA), and nucleotide sequences were analyzed using SnapGene 1.1.3 (Dotmatics, Boston, MA, USA) software to confirm sequence integrity, reading frames, absence of stop codons, and clonal diversity.

### 2.3. Expression and Purification of Target Antibodies

Recombinant VHH antibodies were expressed in *E. coli Rosetta* cells (Novagen, Darmstadt, Germany) transformed with an expression plasmid carrying the target gene under the control of the lac promoter. Cultures were grown in LB medium supplemented with ampicillin (100 µg/mL) and chloramphenicol (34 µg/mL) at 37 °C to an OD_600_ of 0.5–1.0, followed by induction with 1 mM IPTG for 4 h. Cells were harvested, disrupted by sonication, and clarified by centrifugation, and the supernatant was filtered through 0.22 µm PES membranes (Millipore, Darmstadt, Germany). Recombinant antibodies were purified by immobilized metal affinity chromatography on a 5 mL HisTrap FF column using an ÄKTA start system (Cytiva, Marlborough, MA, USA). Antibody purity was evaluated by SDS-PAGE and anti-His immunoblotting, protein concentration was determined by the Lowry assay, and purified samples were dialyzed against phosphate buffer. When required, an additional polishing step was performed by size-exclusion chromatography on a HiPrep 16/60 Sephacryl S-100 HR column (Cytiva, Marlborough, MA, USA).

### 2.4. Enzyme-Linked Immunosorbent Assay

The antigen-binding properties and serotype specificity of the recombinant VHH antibodies were evaluated by sandwich ELISA. VHH antibodies (10 µg/mL) were immobilized on 96-well plates (Corning, Corning, NY, USA) overnight at 4 °C, and nonspecific binding sites were blocked with 1% fetal bovine serum in PBS containing 0.05% Tween-20. A panel of purified D-antigens from Sabin (LSc 2ab type 1, P712 Ch 2ab type 2, and Leon 12a1b type 3) and wild-type (Mahoney, MEF-1, and Saukett) poliovirus strains was used to evaluate serotype specificity and cross-reactivity. H-antigens were generated by heat treatment at 56 °C for 1 h to assess the ability of the antibodies to distinguish native and altered capsid conformations. Antigens were tested from an initial concentration of 50 DU/mL using three-fold serial dilutions and incubated for 2 h at 37 °C. Bound antigen was detected with serotype-specific biotinylated rabbit polyclonal antibodies followed by horseradish peroxidase-conjugated streptavidin. Color development was performed with TMB (EcoTech, Moscow, Russia), and absorbance was measured at 450 nm using a Multiskan FC microplate reader (Thermo Fisher, Waltham, MA, USA). Prior to the final experimental setup, all ELISA components, including the detection antibodies and capture VHHs, were systematically cross-tested to exclude any potential cross-reactivity. The assay configuration was designed to ensure that non-specific binding of the detection antibodies to the capture VHHs or to the plate surface did not contribute to the background signal (Supplementary Figure S4).

### 2.5. Evaluation of the Neutralizing Activity of Recombinant Antibodies

Recombinant VHH antibodies were evaluated for neutralizing activity against Sabin poliovirus types 1–3 and the corresponding wild-type strains Mahoney, MEF-1, and Saukett. Virus titers were determined on Vero cells by endpoint dilution with visual assessment of cytopathic effect (CPE) in accordance with WHO recommendations [29].

Cells were seeded in 96-well plates at 3 × 10^4^ cells per well and cultured in DMEM containing 5% FBS, 2 mM L-glutamine, penicillin (100 U/mL), and streptomycin (100 µg/mL) until a 90–95% monolayer formed. Ten-fold virus dilutions (10^−1^–10^−8^) were prepared in DMEM with 2% FBS and tested in eight replicates. CPE was recorded after 5–7 days, and virus titers were calculated using the Kärber method [30].

For neutralization, viruses were adjusted to 50 TCID_50_/well and mixed 1:1 with two-fold serial dilutions of VHH antibodies starting at 50 µg/mL. After incubation for 1 h at 37 °C, the mixtures were added to Vero cell monolayers. Cell, virus, and antibody-toxicity controls were included, and virus back-titration was performed in parallel. Cultures were monitored daily for 7 days at 37 °C and 5% CO_2_. The IC_50_ was defined as the VHH concentration that protected 50% of replicate wells from virus-induced cytopathic effect and was calculated using the Kärber method.

### 2.6. Statistical Analysis

**Figure 4:**
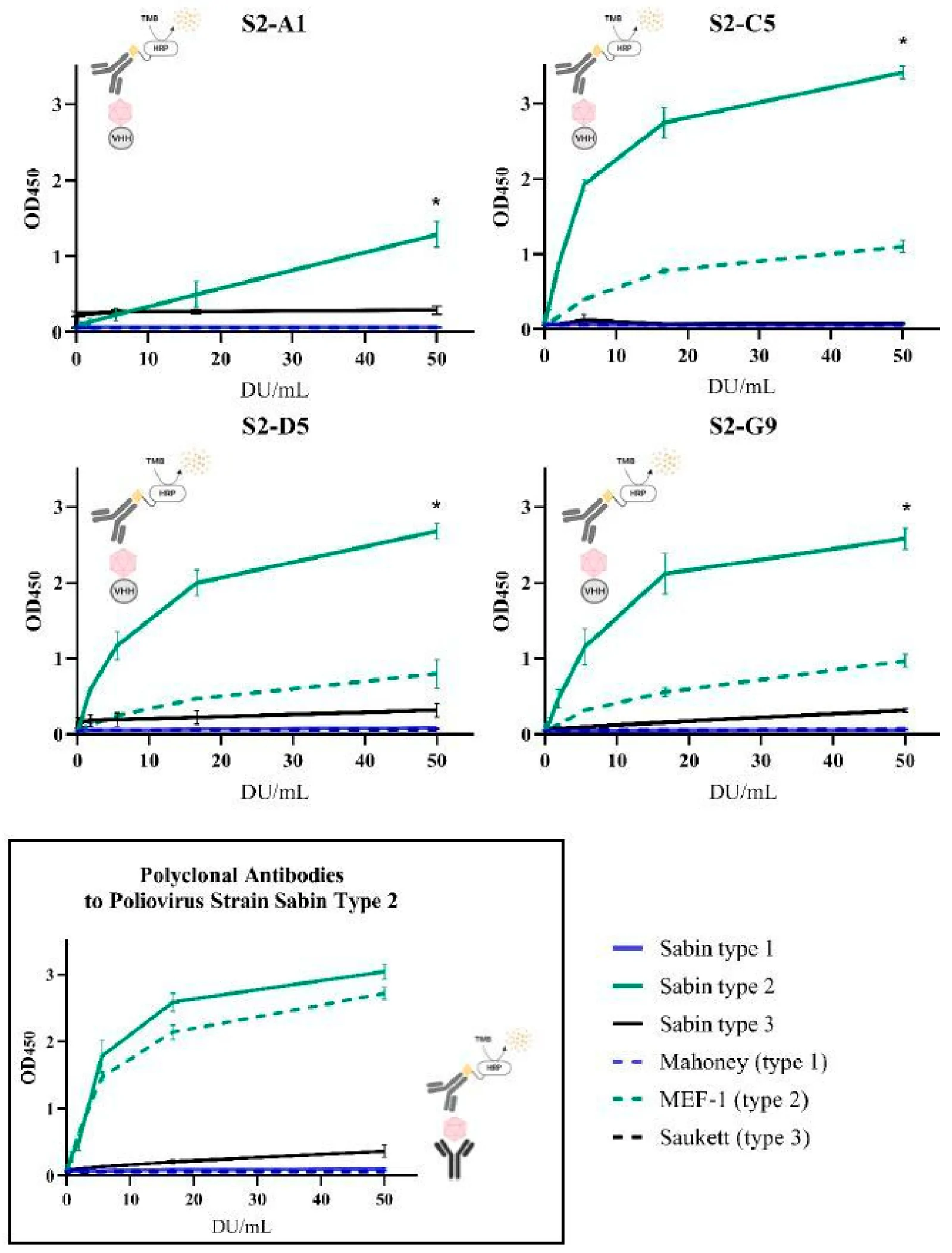
Analysis of nanobody specificity against poliovirus Sabin type 2 by ELISA in comparison with a polyclonal antibody-based test system. The graphs show the dependence of optical density (OD_450_) on D-antigen concentration. Blue solid line–Sabin type 1 D-antigen; green solid line–Sabin type 2 D-antigen; black solid line–Sabin type 3 D-antigen. Blue dashed line–Mahoney D-antigen (type 1); green dashed line–MEF-1 D-antigen (type 2); and black dashed line–Saukett D-antigen (type 3). Each nanobody against Sabin type 2 (S2-A1; S2-C5; S2-D5; S2-G9) was coated onto the plate bottom at a concentration of 10 µg/mL. Polyclonal antibodies were used at a working dilution of 1/500, determined empirically. Data are shown as the mean values and standard deviation (SD) from three independent experiments (*n* = 3). * *p* < 0.05 (Tukey’s multiple comparisons test) indicates a statistically significant difference between the binding of the given VHH to the homologous Sabin type 2 D-antigen and its binding to heterologous antigens (Sabin type 1, Sabin type 3, Mahoney, MEF-1, Saukett) at the same D-antigen concentration.

**Figure 5:**
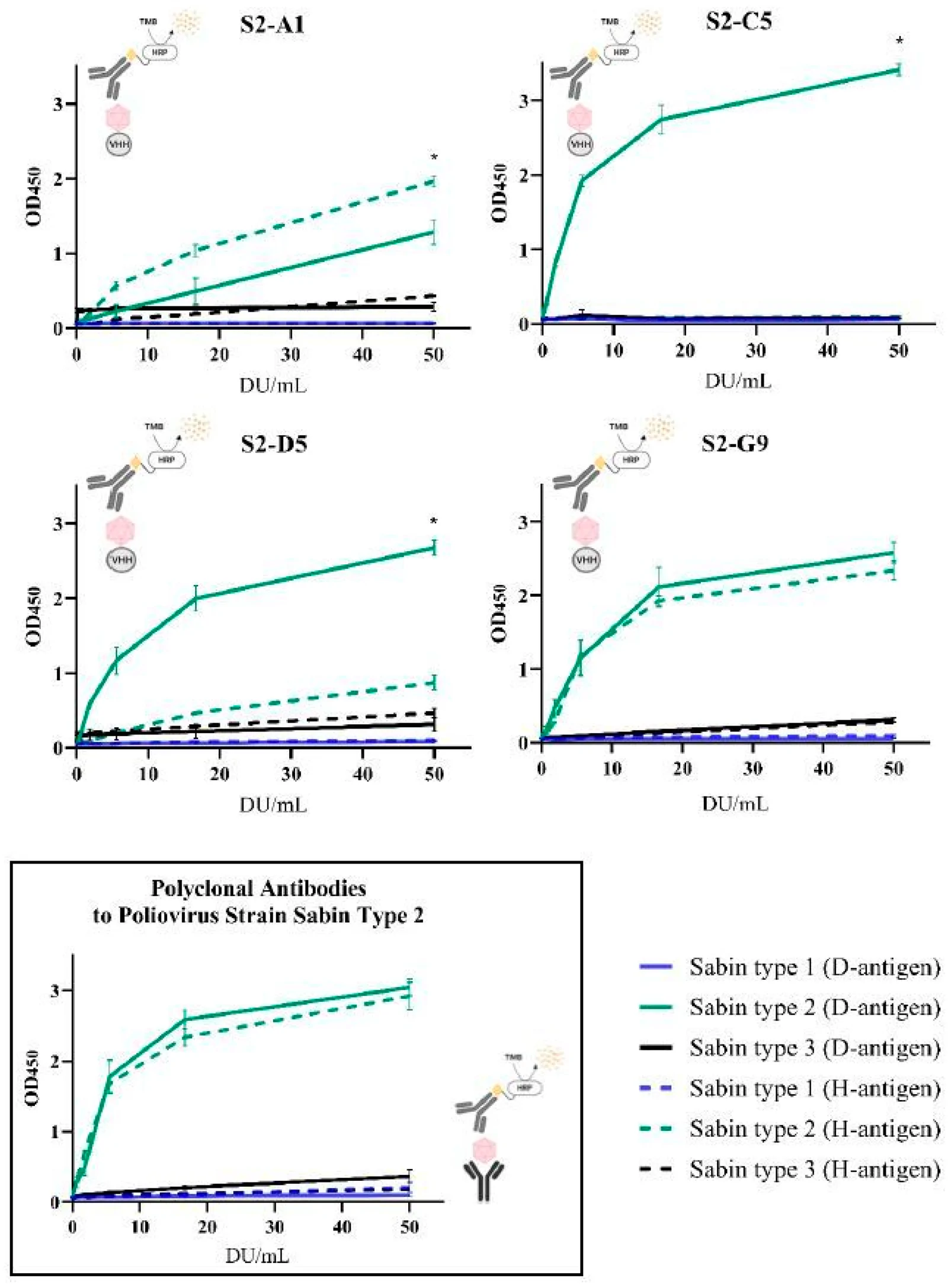
Analysis of D- and H-antigen differentiation of poliovirus Sabin type 2 by nanobodies and a polyclonal system using ELISA. The graphs show the dependence of optical density (OD_450_) on antigen concentration (DU/mL). Solid lines–D-antigen; dashed lines–H-antigen (obtained by heat treatment at 56 °C, 1 h). Green lines–Sabin type 2 antigen. Each nanobody against Sabin type 2 (S2-A1; S2-C5; S2-D5; S2-G9) was coated onto the plate bottom at a concentration of 10 µg/mL. Polyclonal antibodies were used at a working dilution of 1/500, determined empirically. Data are shown as the mean values and standard deviation (SD) from three independent experiments (*n* = 3). * *p* < 0.05 (Tukey’s multiple comparisons test) indicates a statistically significant difference between binding to the D-antigen and binding to the H-antigen at the same antigen concentration.

**Figure 6:**
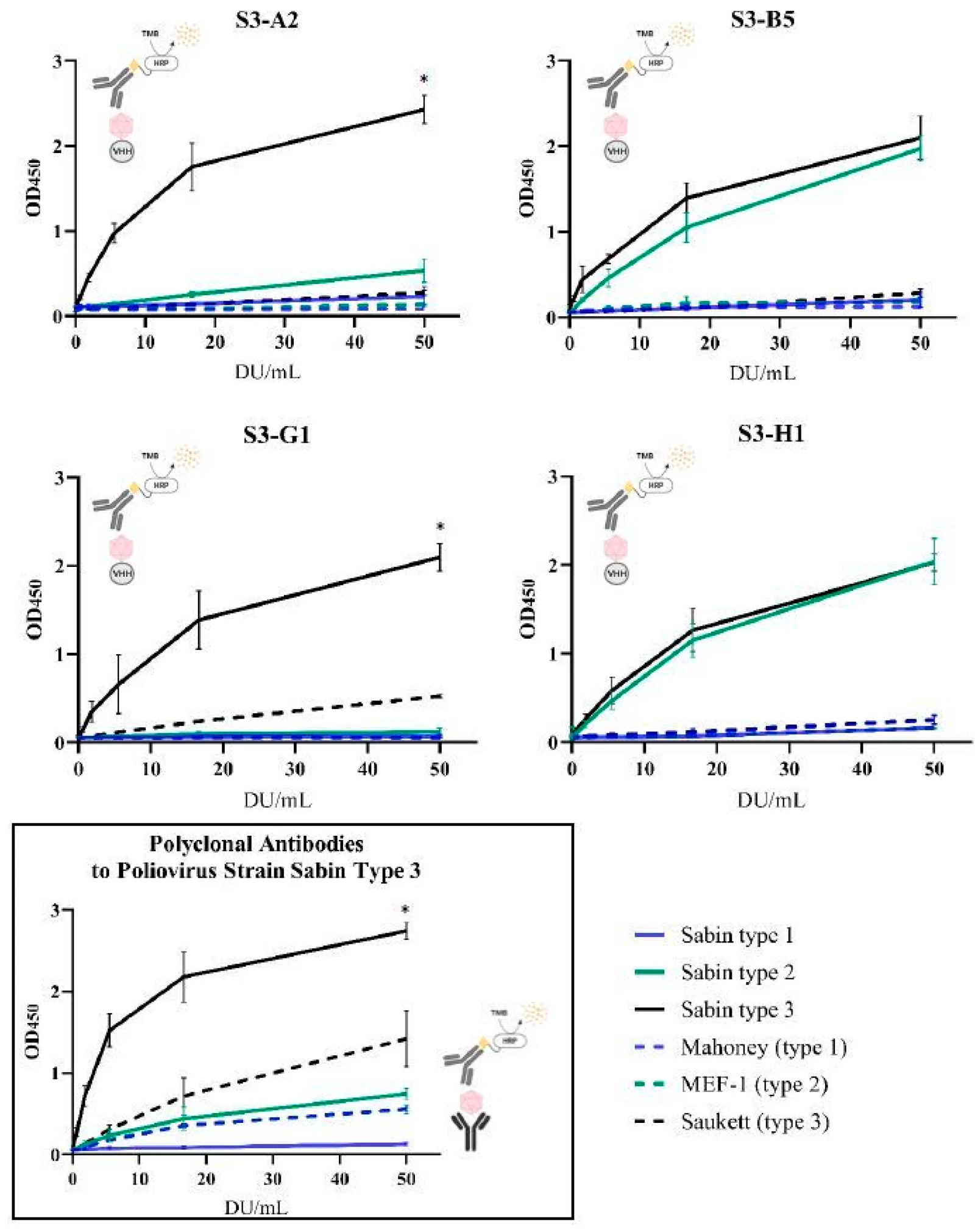
Analysis of nanobody specificity against poliovirus Sabin type 3 by ELISA in comparison with a polyclonal antibody-based test system. The graphs show the dependence of optical density (OD_450_) on D-antigen concentration. Blue solid line–Sabin type 1 D-antigen; green solid line–Sabin type 2 D-antigen; and black solid line–Sabin type 3 D-antigen. Blue dashed line–Mahoney D-antigen (type 1); green dashed line–MEF-1 D-antigen (type 2); black dashed line–Saukett D-antigen (type 3). Each nanobody against Sabin type 3 (S3-A2; S3-B5; S3-G1; S3-H1) was coated onto the plate bottom at a concentration of 10 µg/mL. Polyclonal antibodies were used at a working dilution of 1/500, determined empirically. Data are shown as the mean values and standard deviation (SD) from three independent experiments (*n* = 3). * *p* < 0.05 (Tukey’s multiple comparisons test) indicates a statistically significant difference between the binding of the given VHH to the homologous Sabin type 3 D-antigen and its binding to heterologous antigens (Sabin type 1, Sabin type 2, Mahoney, MEF-1, Saukett) at the same D-antigen concentration.

**Figure 7:**
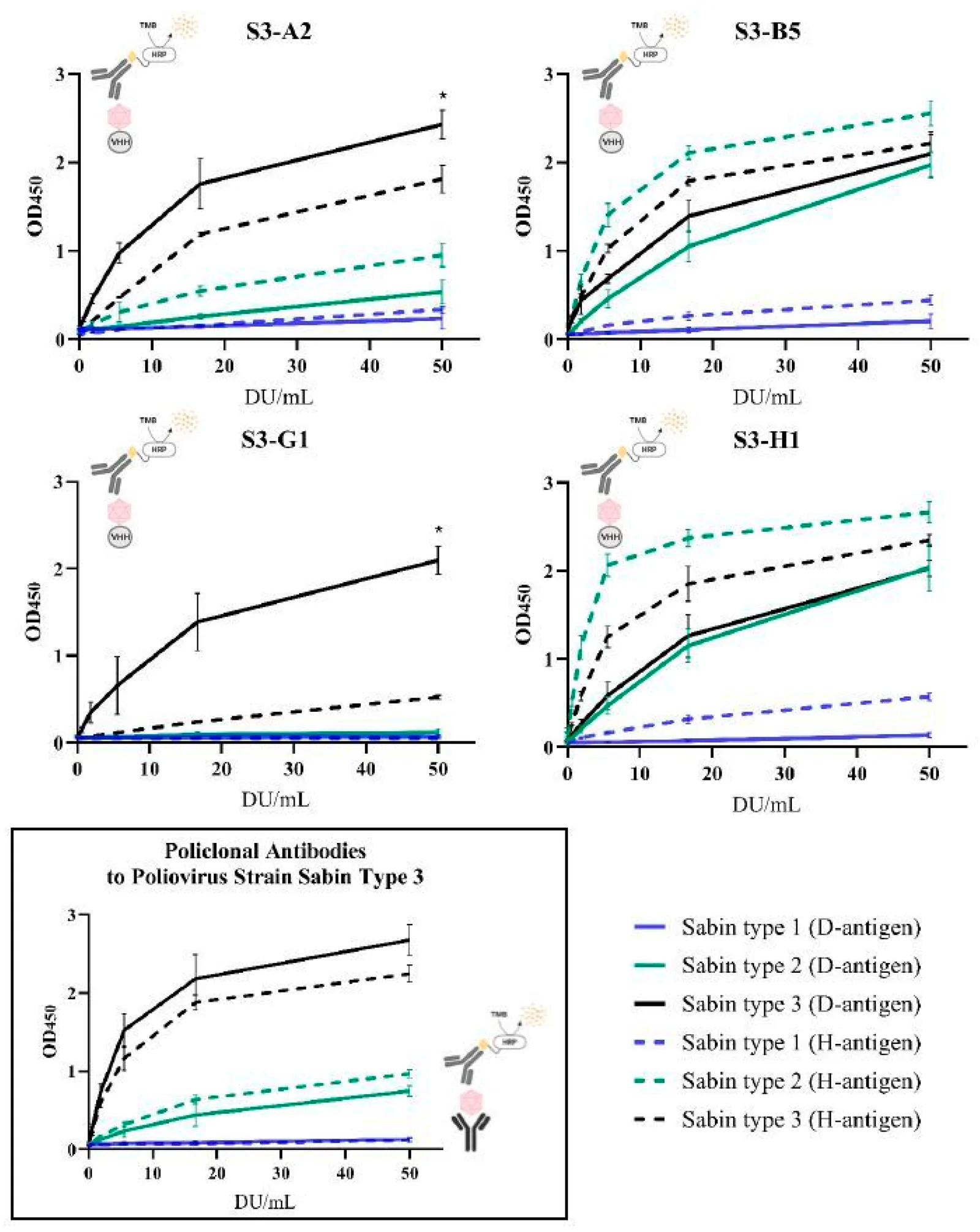
Analysis of D- and H-antigen differentiation of poliovirus Sabin type 3 by nanobodies and a polyclonal system using ELISA. The graphs show the dependence of optical density (OD_450_) on antigen concentration (DU/mL). Solid lines–D-antigen; dashed lines–H-antigen (obtained by heat treatment at 56 °C, 1 h). Black lines–Sabin type 3 antigen. Each nanobody against Sabin type 3 (S3-A2; S3-B5; S3-G1; S3-H1) was coated onto the plate at a concentration of 10 µg/mL. Polyclonal antibodies were used at a working dilution of 1/500, determined empirically. Data are shown as the mean values and standard deviation (SD) from three independent experiments (*n* = 3). * *p* < 0.05 (Tukey’s multiple comparisons test) indicates a statistically significant difference between binding to the D-antigen and binding to the H-antigen at the same antigen concentration.

Statistical analysis and data visualization were performed using GraphPad Prism 8.0 software (GraphPad Software, Boston, MA, USA). All experiments were conducted in three independent replicates (*n* = 3). Data are shown as mean ± SD. Normality of data distribution was assessed using the Shapiro–Wilk test. Two-way ANOVA was applied using two independent factors appropriate for each experimental design: poliovirus serotype/strain (Sabin type 1, Sabin type 2, Sabin type 3, Mahoney, MEF-1, Saukett) and D-antigen concentration (DU/mL) for the specificity experiments (Figure 4 and Figure 6), and antigenic form (D versus H) and antigen concentration (DU/mL) for the differentiation experiments (Figure 5 and Figure 7), with the main effects of each factor and their interaction assessed in both models. For multiple comparisons between groups, Tukey’s post hoc test was applied. Differences were considered statistically significant at *p* < 0.05.

## 3. Results

### 3.1. Immunization of Alpacas and Determination of Immune Serum Titers

To induce an immune response, alpacas were immunized with purified poliovirus D-antigens obtained from attenuated Sabin strains types 2 and 3. Analysis of blood samples showed an increase in antigen-specific antibody titers after the third immunization. Specifically, serum from the first animal immunized with poliovirus type 2 exhibited a titer of 1:1024, while serum from the second animal immunized with type 3 antigen reached a titer of 1:2048. No specific antibodies were detected in pre-immune sera.

### 3.2. Construction of Phage Libraries

Five days after the final immunization, 100 mL of whole blood was collected for PBMC isolation and phage library preparation. The size of each library was determined: 5 × 10^7^ CFU/mL–for Sabin type 2, while that against type 3 was approximately 6 × 10^7^ CFU/mL. For the primary round of biopanning, transformed *E. coli* TG1 cells were infected with M13KO7 helper phage. The resulting phage display libraries had titers of approximately 10^13^ PFU/mL.

### 3.3. Selection and Screening of Antibodies

**Figure 1:**
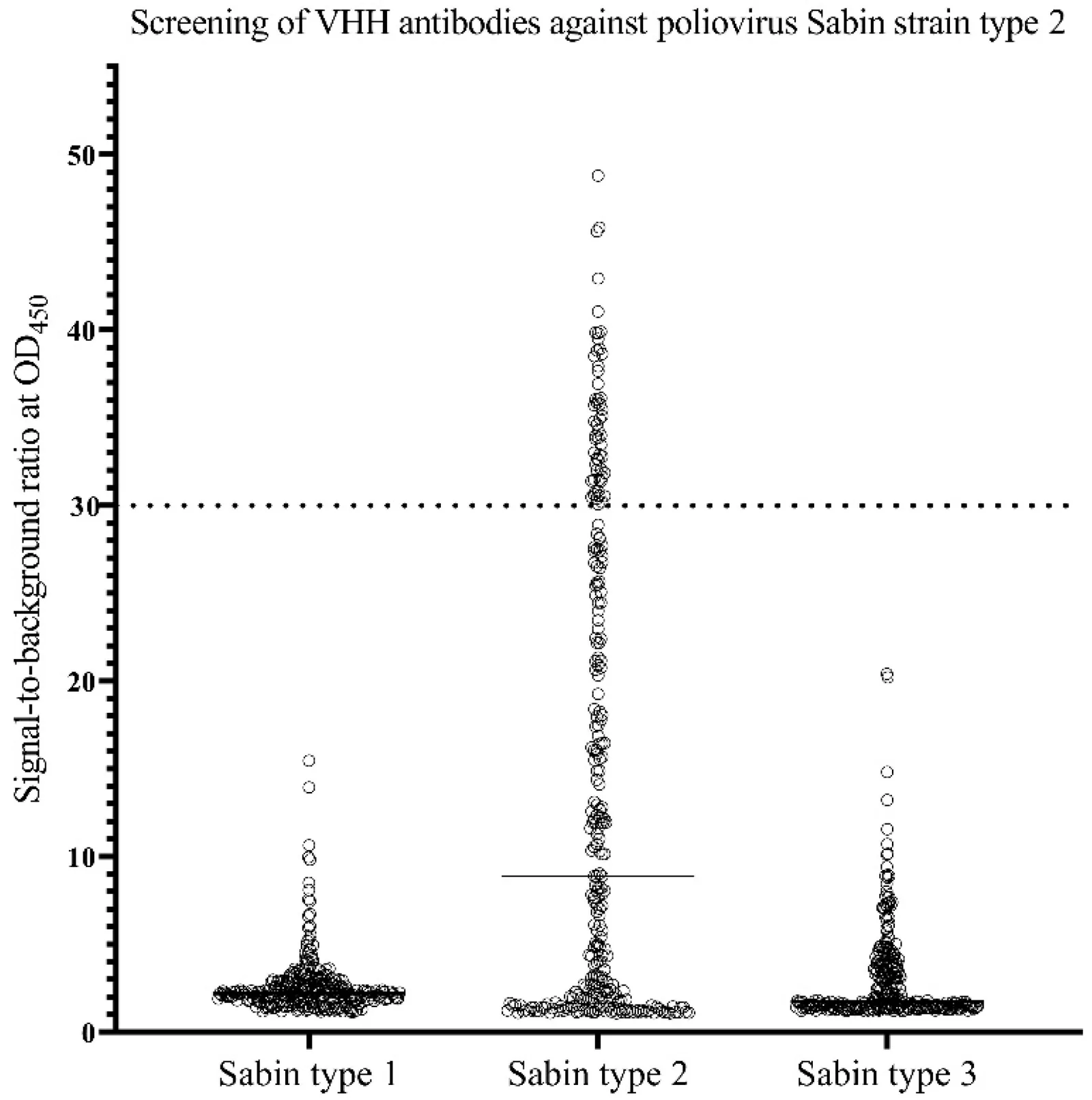
Screening of the VHH antibody library against poliovirus Sabin strain type 2 by ELISA. The optical density (OD_450_) of each sample was normalized to the background value (0.055), and the signal-to-background ratio was calculated. Data are shown as individual values for each clone. The selection threshold was set at a signal-to-background ratio ≥ 30. Clones with a ratio above this value were selected for further analysis.

**Figure 2:**
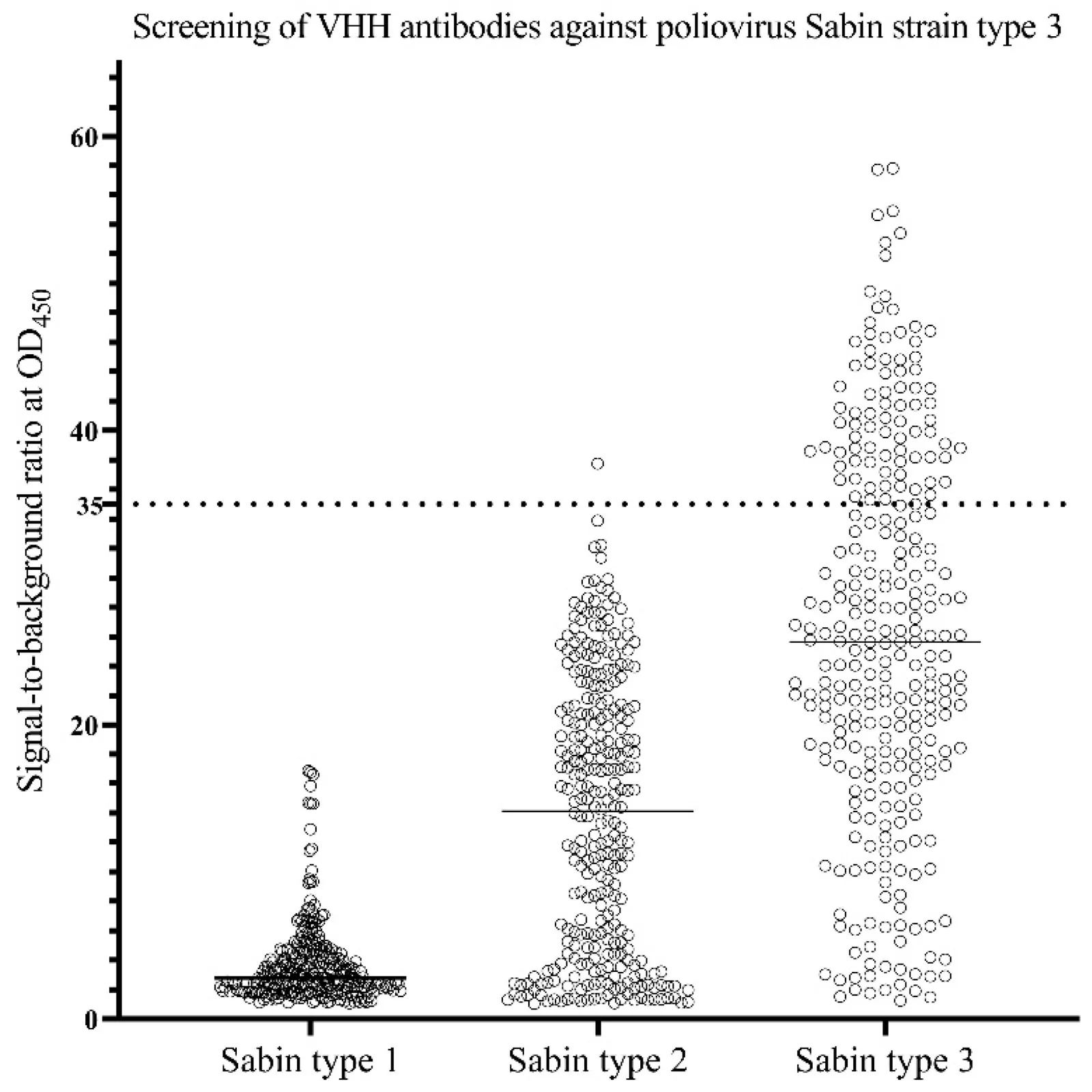
Screening of the VHH antibody library against poliovirus Sabin strain type 3 by ELISA. The optical density (OD_450_) of each sample was normalized to the background value (0.055), and the signal-to-background ratio was calculated. Data are shown as individual values for each clone. The selection threshold was set at a signal-to-background ratio ≥ 35. Clones with a ratio above this value were selected for further analysis.

The obtained phage libraries were used for biopanning—a cyclic selection process of phage particles specifically binding to the target poliovirus D-antigen, followed by amplification of eluted clones to enrich the library for antigen-binding clones. After three rounds of affinity selection, individual nanobody clones were screened by ELISA. For the library against poliovirus type 2, 56 out of 288 analyzed clones (19.4%) showed a ≥30-fold increase in the signal-to-background ratio upon binding to the target antigen (Figure 1). For the library against type 3, the number of positive clones was 82 out of 288 (28.5%), with a selection threshold of ≥35 (Figure 2). The selection threshold for each library was set individually depending on the number of clones with desired properties and the optical density signal (OD_450_).

During screening, VHHs were analyzed for binding to the target–purified poliovirus antigen of the Sabin strain (type 2 or 3)—and were also tested for interaction with the two other serotypes. Positive clones showed no cross-reactivity with related poliovirus serotypes or with negative control samples (5% bovine serum albumin (BSA), purified inactivated SARS-CoV-2 virus at 50 µg/mL). All specific clones were selected for Sanger sequencing of the VHH variable domains and identification of unique CDR regions. Sequences were selected based on the expected fragment size (110–130 amino acids) for single-domain antibodies [31]. Amino acid sequence analysis of the recombinant VHH antibodies allowed the selection of four clones against poliovirus Sabin type 2 and five clones against type 3. One of the five Sabin type 3 candidates was excluded from the panel reported here because it demonstrated cross-reactivity with other poliovirus types. The final panel presented in this study therefore consisted of four clones against poliovirus Sabin type 2 and four against poliovirus Sabin type 3 (Supplementary Figures S5 and S6).

### 3.4. Expression and Purification of Target Antibodies

**Figure 3:**
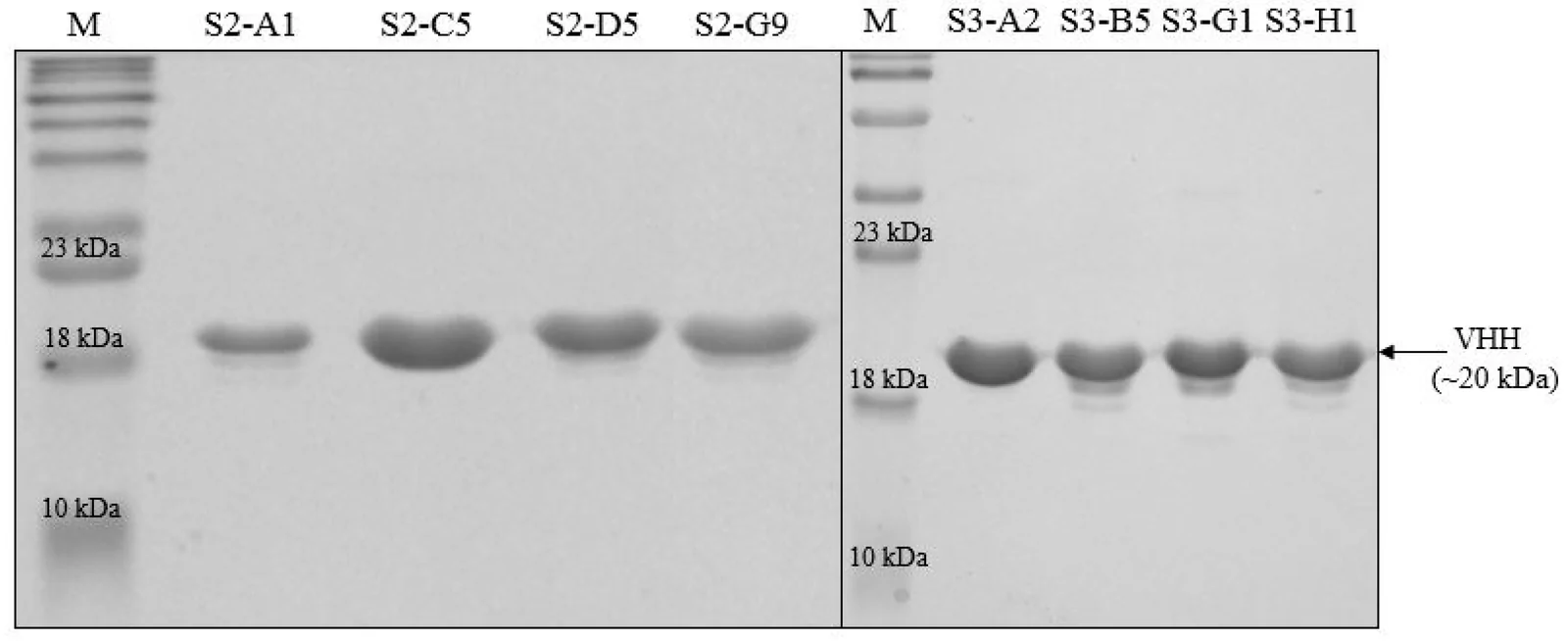
Analysis of purified antibodies by electrophoresis in 20% polyacrylamide gel stained with Coomassie R-250. Left: pool of VHHs (S2-A1, S2-C5, S2-D5, S2-G9) against poliovirus Sabin type 2 strain. Right: pool of VHHs (S3-A2, S3-B5, S3-H1, S3-G1) against poliovirus Sabin type 3 strain. The molecular weight of the target protein is ~20 kDa. Center: Prestained Protein Marker II (10–200 kDa) (Servicebio, China).

To confirm the antigen-binding activity of the obtained antibodies in the absence of phage particles, the selected clones were expressed in *E. coli* Rosetta CodonPlus cells with 1 mM IPTG induction and purified from cell lysates by immobilized metal affinity chromatography (IMAC) using Ni-NTA resin. To remove imidazole and exchange the buffer, dialysis against PBS was performed, followed by concentration of the samples using centrifugal filters with a 10 kDa molecular weight cutoff. Electrophoretic analysis results demonstrated successful purification of monomeric VHH antibodies with the expected molecular weight of approximately 20 kDa (Figure 3).

When the purity of the antibody preparations was not satisfactory, an additional size-exclusion chromatography purification step was performed.

### 3.5. Evaluation of Antibody Neutralizing Activity

To evaluate the neutralizing activity of the generated VHH antibodies, individual nanobodies selected against Sabin poliovirus types 2 and 3 were tested in a Vero cell-based neutralization assay using attenuated Sabin strains (types 1–3) and the corresponding wild-type strains (Mahoney, MEF-1, and Saukett). The working virus dose was 50 TCID_50_/well. Prior to the assay, the highest non-cytotoxic concentration of each antibody was determined in Vero cells and used as the starting concentration for two-fold serial dilutions. No endotoxin was detected in the purified antibody preparations using the LAL assay [32]. Antibody concentrations were determined by the Lowry method.

Three VHHs (S2-C5, S2-G9, S2-D5) specifically neutralized poliovirus Sabin type 2 and showed no neutralizing activity against the homologous wild-type MEF-1 strain or other serotypes. The highest neutralizing activity was demonstrated by clone S2-C5, at a concentration of 1.06 µg/mL (IC_50_ = 55.8 nM). Clones S2-G9 and S2-D5 neutralized the virus at concentrations of 1.10 and 4.42 µg/mL, respectively (Table 1).

Thus, three clones against type 2 possess neutralizing activity, although their efficacy varies over a 4-fold concentration range. The neutralizing activity of S2-C5, S2-G9 and S2-D5 clones against Sabin 2 poliovirus suggests that the selected antibodies may interfere with one or more steps of the infectious process although the specific mechanism remains to be determined.

All tested VHH against Sabin 3 poliovirus did not display virus-neutralizing activity.

### 3.6. Analysis of the Specificity of Nanobodies Against Sabin Type 2 by ELISA

The antigen-binding activity of four nanobodies selected against poliovirus Sabin type 2 (S2-A1, S2-C5, S2-D5, and S2-G9) was assessed by ELISA (Figure 4). In this assay, VHH nanobodies were used as capture antibodies, whereas biotinylated polyclonal antibodies against Sabin type 2 served as the detection antibodies. The purified inactivated D-antigens of three attenuated Sabin strains (types 1, 2, and 3) and three wild-type poliovirus strains (Mahoney, MEF-1, and Saukett) were used as antigens. The initial D-antigen concentration (DU/mL) for all viruses was 50 DU/mL and had been previously determined by ELISA using rabbit polyclonal antibodies [28] and NIBSC international standard 17/160 obtained from WHO [14].

All four VHHs, when used as capture reagents, demonstrated pronounced specificity for the D-antigen of the Sabin type 2 vaccine strain. For nanobodies S2-C5, S2-D5, and S2-G9, moderate cross-reactivity with the homologous wild-type MEF-1 strain (OD_450_ ≤ 1) was observed. In control experiments with polyclonal antibodies against Sabin type 2 used as capture reagents, specific binding to the target antigen was also confirmed. However, cross-reactivity with wild-type strains was more pronounced.

### 3.7. Analysis of the Ability of Nanobodies to Differentiate Between D- and H-Antigens of Poliovirus by ELISA Among VHHs Selected Against Sabin Type 2

The ability of the selected VHHs to distinguish the native D-antigen from its non-immunogenic H-form was assessed by ELISA. Purified Sabin type 2 D-antigen was adjusted to 50 DU/mL and divided into two equal aliquots. One aliquot was kept untreated (D-antigen), whereas the second was incubated at 56 °C for 1 h to convert the D-antigen to the H-antigen. In parallel, polyclonal antibodies against Sabin type 2 were tested under the same conditions for comparative evaluation of the specificity of the two systems. The results are presented in Figure 5.

In the D- and H-antigen differentiation experiment by ELISA, monoclonal antibodies S2-C5 and S2-D5 specifically recognized the D-antigen, indicating their ability to recognize a conformational epitope characteristic of the mature viral particle. The presence of such clones opens prospects for developing a test system free from cross-reactivity with the H-form. In contrast, nanobody S2-G9 interacted with both antigen forms, demonstrating a profile similar to that of polyclonal antibodies. Notably, nanobody S2-A1 demonstrated preferential binding to the H-antigen relative to the D-antigen.

### 3.8. Analysis of Nanobody Specificity Against Sabin Type 3 by ELISA

The antigen-binding activity of four VHHs selected against poliovirus Sabin type 3 (S3-A2, S3-B5, S3-G1, and S3-H1) was assessed by ELISA (Figure 6) following a scheme analogous to that used for type 2.

When analyzing the specificity of four VHHs selected against poliovirus Sabin type 3 (S3-A2, S3-B5, S3-G1, and S3-H1) by ELISA, nanobodies S3-A2 and S3-G1 demonstrated high serotype specificity, recognizing exclusively the D-antigen of the homologous strain and showing no interaction with antigens of other serotypes. In contrast, nanobodies S3-B5 and S3-H1, while not binding to wild-type strains, exhibited cross-reactivity with the Sabin type 2 D-antigen. Polyclonal antibodies against type 3 under the same conditions were characterized by a broader binding spectrum.

### 3.9. Analysis of the Ability of Nanobodies to Differentiate Between D- and H-Antigens of Poliovirus by ELISA Among VHHs Selected Against Sabin Type 3

To assess the ability of VHHs generated against poliovirus Sabin type 3 to distinguish between the native virion and H-antigen, an ELISA was performed. Purified Sabin type 3 D-antigen was adjusted to 50 DU/mL and divided into two equal aliquots. One aliquot was kept untreated (D-antigen), whereas the second was incubated at 56 °C for 1 h to convert the D-antigen to the H-antigen. For comparative specificity evaluation, polyclonal antibodies against the same strain were tested under identical conditions. The results are presented in Figure 7.

In the D- and H-antigen differentiation experiment by ELISA among the four tested VHHs (S3-A2, S3-B5, S3-G1, and S3-H1), nanobody S3-G1 specifically recognized only the D-antigen, showing no interaction with the H-form, when used as capture antibody. Nanobody S3-A2 also exhibited preferential binding to the D-antigen; however, it demonstrated significant, albeit lower, interaction with the H-antigen (OD_450_ ≤ 2). Nanobodies S3-B5 and S3-H1 bound both antigen forms without preferential recognition of either form. Polyclonal antibodies against Sabin type 3 also failed to distinguish between the D- and H-forms of the homologous strain and, in addition, moderately interacted with Sabin type 2 D- and H-antigens (OD_450_ ≤ 1).

Thus, nanobody S3-G1 exhibits the highest specificity for the Sabin type 3D-antigen among the tested antibody preparations.

## 4. Discussion

The present study demonstrates the feasibility of generating recombinant VHH anti-bodies against the D-antigen of poliovirus Sabin types 2 and 3. Previously, nanobodies against these serotypes had not been described, with available studies predominantly focused on fundamental investigation of neutralization mechanisms of poliovirus type 1 [24,25]. Together with our previous work on Sabin type 1, these findings indicate that highly specific VHH reagents can now be obtained against all three Sabin serotypes, providing a foundation for the development of recombinant antibody-based analytical tools for inactivated poliovirus vaccine quality control. However, their performance in actual vaccine preparations or production intermediates, as well as their suitability within a validated quantitative assay, remain to be established in future studies. In the present study, several nanobodies exhibited substantially narrower specificity than the corresponding polyclonal antibodies, supporting the concept that recombinant VHHs may improve assay selectivity when incorporated into existing ELISA formats as capture antibodies while retaining polyclonal antibodies for detection.

Phage display selection from alpaca immune libraries enabled the generation of specific antibodies against both serotypes; however, the selection efficiency and properties of the obtained nanobodies differed. For serotype 2, screening of 288 clones after three rounds of selection (Figure 1) allowed the isolation of an antibody pool with high serotype specificity and neutralizing activity. For serotype 3, selection was complicated by the fact that clones specific to type 3 also showed interaction with the Sabin type 2D-antigen, hindering the isolation of strictly specific clones (Figure 2). Such cross-reactivity is likely due to the high degree of structural homology of antigenic sites on the capsid surface of serotypes 2 and 3, as previously noted in the literature [17,33].

Four nanobodies selected against serotype 2 demonstrated high specificity in ELISA, when used as capture immunosorbent. The selected VHHs showed a clear advantage in specificity towards vaccine Sabin 2 strain compared to the wild-type strain. Moderate cross-reactivity with the MEF-1 antigen (OD_450_ ≤ 1) was observed, which may be attributed to the high degree of structural similarity of capsid proteins between the vaccine and wild-type viruses within the same serotype [34,35].

Three VHH antibodies demonstrated different levels of neutralizing activity against Sabin 2 poliovirus in a Vero cell-based system. VHH S2-C5 showed IC_50_ of 1.06 µg/mL, corresponding to 55.8 nM. VHH S2-G9 showed a comparable activity with an IC_50_ of 1.10 µg/mL (58.2 nM). In contrast, VHH S2-D5 demonstrated lower neutralizing potency, with IC_50_ of 4.42 µg/mL (232.6 nM). Thus, the IC_50_ value of S2-D5 was approximately fourfold higher than those of S2-C5 and S2-G9, indicating lower functional neutralizing potency under the conditions of the assay. These differences may reflect variation in epitope recognition. In particular, a VHH targeting an epitope located within or near a functionally critical receptor-binding region may neutralize Sabin 2 more efficiently than an antibody binding to a less functionally important or less accessible site.

When analyzing the ability of the nanobodies to differentiate between D- and H-antigens (Figure 5), two nanobodies (S2-C5 and S2-D5) demonstrated specific binding to the native capsid form with residual or no interaction with the H-form. In contrast, nanobody S2-A1 exhibited preferential binding to H-antigen. Thus, the antibody panel against type 2 has the potential to enable monitoring of both D-antigen content and H-antigen impurity levels in vaccine preparations.

When analyzing the specificity of four nanobodies selected against poliovirus Sabin type 3 by ELISA (Figure 6), two antibodies (S3-A2 and S3-G1) demonstrated high serotype specificity, recognizing exclusively the D-antigen of the homologous strain, whereas the other two (S3-B5 and S3-H1) exhibited cross-reactivity with the Sabin type 2 D-antigen. This distribution of properties may be related to the selection conditions during screening (Figure 2), where the presentation of clones as phage-displayed fusion proteins could have influenced their specificity. It is known that within the phage particle, the conformation of the antibody and the accessibility of its antigen-binding site differ from those in the free form. Upon elimination of the phage p3 protein, to which VHHs are fused during display, conformational constraints are relieved, which may lead to changes in specificity [36,37]. We hypothesize that this explains why clones were selected as specific to type 3 during phage screening but after expression as soluble proteins, acquired the ability to also bind the type 2 antigen. In the experiment assessing the ability of VHHs to differentiate between poliovirus D- and H-antigens (Figure 7), nanobody S3-G1 specifically recognized only the D-antigen, without interacting with the non-immunogenic H-form. Nanobody S3-G1 was the only clone that combined high serotype specificity, absence of interaction with wild-type strains, and the ability to discriminate between D- and H-antigens, making it the most promising reagent among the tested VHHs against Sabin 3 poliovirus.

Polyclonal sera recognize multiple epitopes distributed across the viral capsid, providing broad antigen recognition. While this feature facilitates antigen detection, it may reduce the ability to distinguish closely related antigenic forms. For tasks requiring high discriminatory ability, such as distinguishing between vaccine and wild-type strains or between D- and H-antigen forms, monoclonal nanobodies (S2-C5 and S2-D5 for type 2 and S3-G1 for type 3) demonstrate a clear advantage due to their narrow specificity and absence of cross-reactions. At the same time, it should be noted that polyclonal antibodies also show appreciable specificity for the D-antigen, although they are less selective than the VHH reagents [28]. Therefore, a combined approach using both polyclonal and VHH antibodies may offer improved discrimination between D- and H-antigen forms, potentially enhancing the accuracy and reliability of immunoassays for vaccine quality control.

A promising direction for further research is the construction of full-length antibodies based on the obtained VHH sequences, including in the VHH-Fc format [38,39]. The increased avidity provided by Fc-mediated dimerization may further enhance antigen binding and improve assay sensitivity. This enhanced avidity could improve the stability of the antibody-virus complex and potentially increase neutralization activity. Such antibodies could therefore expand the utility of these VHHs in analytical applications.

The obtained VHHs represent highly specific recombinant reagents for poliovirus antigen characterization, complementing polyclonal sera and offering potential for the development of standardized assays requiring precise differentiation of antigenic forms.

### Research Limitations

This study has several limitations. First, the epitopes recognized by the selected VHHs were not mapped, which prevents thorough prediction of their ability to recognize and neutralize antigenic variants of Sabin 2 poliovirus. In addition, since the screening library was generated from an immunized alpaca, its sequence diversity may not fully represent the broader repertoire of potentially neutralizing VHHs. Furthermore, the VHH-Fc format would be a natural direction of antibody development.

## 5. Conclusions

To our knowledge, this is the first study to generate and characterize panels of recombinant VHH antibodies raised against the D-antigens of poliovirus Sabin types 2 and 3. Several nanobodies exhibited high serotype specificity. Although some clones also recognized the H-antigen, selected clones discriminated between the D- and H-antigens, demonstrating their potential to improve the specificity of poliovirus antigen detection. These recombinant antibodies provide valuable reagents for the development of next-generation immunoassays for inactivated poliovirus vaccine quality control and represent a foundation for further engineering of VHH-based antibody formats.

## Figures and Tables

**Table 1:** Neutralizing activity of VHH clones against Sabin 2 poliovirus in Vero-cell based assay.

VHH	IC_50,_ µg/mL	IC_50,_ nM
S2-C5	1.06	55.8
S2-G9	1.10	58.2
S2-D5	4.42	232.6

## Data Availability

Raw data are available from the authors upon reasonable request.

## Supplementary Materials

The following supporting information can be downloaded at https://www.mdpi.com/article/10.3390/v18101147/s1: Supplementary Figure S1. SDS-PAGE gel showing purification of recombinant VHHs: S2-A1, S2-C5, S2-D5, S2-G9. Supplementary Figure S2. SDS-PAGE gel showing purification of recombinant VHHs: S3-A2, S3-B5, S3-H1, S3-G1. Supplementary Figure S3. Antigenic structure of poliovirus. Supplementary Figure S4. Raw ELISA data demonstrating the absence of cross-reactivity between capture VHH antibodies and detection polyclonal antibodies. Supplementary Figure S5. Multiple sequence alignment of the amino acid sequences of nanobody clones selected against poliovirus Sabin type 2, generated with CLC Sequence Viewer (QIAGEN). Supplementary Figure S6. Multiple sequence alignment of the amino acid sequences of nanobody clones selected against poliovirus Sabin type 3, generated with CLC Sequence Viewer (QIAGEN).

## References

[1] Minor P.D., Martín J. (2016). An Introduction to Poliovirus: Pathogenesis, Vaccination, and the Endgame for Global Eradication. Poliovirus.

[2] World Health Organization (2019). Two Out of Three Wild Poliovirus Strains Eradicated.

[3] Kalkowska D.A., Pallansch M.A., Cochi S.L., Thompson K.M. (2021). Updated Characterization of Poliovirus Transmission in Pakistan and Afghanistan and the Impacts of Different Outbreak Response Vaccine Options. J. Infect. Dis..

[4] Kalkowska D.A., Pallansch M.A., Wassilak S.G.F., Cochi S.L., Thompson K.M. (2023). Serotype 2 oral poliovirus vaccine (OPV2) choices and the consequences of delaying outbreak response. Vaccine.

[5] Platt L.R., Estívariz C.F., Sutter R.W. (2014). Vaccine-Associated Paralytic Poliomyelitis: A Review of the Epidemiology and Estimation of the Global Burden. J. Infect. Dis..

[6] Macklin G., Diop O.M., Humayun A., Shahmahmoodi S., El-Sayed Z.A., Triki H., Rey G., Avagyan T., Grabovac V., Jorba J. (2020). Update on Immunodeficiency-Associated Vaccine-Derived Polioviruses—Worldwide, July 2018–December 2019. MMWR Morb. Mortal. Wkly. Rep..

[7] World Health Organization (2021). Polio Eradication Strategy 2022–2026: Executive Summary.

[8] Beale A.J., Mason P.J. (1962). The measurement of the D-antigen in poliovirus preparations. J. Hyg..

[9] Hogle J.M. (2002). Poliovirus cell entry: Common structural themes in viral cell entry pathways. Annu. Rev. Microbiol..

[10] Lentz K.N., Smith A.D., Geisler S.C., Cox S., Buontempo P., Skelton A., DeMartino J., Rozhon E., Schwartz J., Girijavallabhan V. (1997). Structure of poliovirus type 2 Lansing complexed with antiviral agent SCH48973: Comparison of the structural and biological properties of the three poliovirus serotypes. Structure.

[11] Jacobson M.F., Baltimore D. (1968). Morphogenesis of poliovirus. J. Mol. Biol..

[12] Le Bouvier G.L. (1959). The D to C change in poliovirus particles. Br. J. Exp. Pathol..

[13] Tano Y., Shimizu H., Martin J., Nishimura Y., Simizu B., Miyamura T. (2007). Antigenic characterization of a formalin-inactivated poliovirus vaccine derived from live-attenuated Sabin strains. Vaccine.

[14] World Health Organization (2022). WHO/BS/2022.2432: Report on the WHO Collaborative Study to Establish Universal Reagents for the D-Antigen Potency Testing of Inactivated Polio Vaccines.

[15] Sawyer L.A., McInnis J., Albrecht P. (1993). Quantitation of D antigen content in inactivated poliovirus vaccine derived from wild-type or sabin strains. Biol. J. Int. Assoc. Biol. Stand..

[16] Minor P.D., Racaniello V.R. (1990). Antigenic Structure of Picornaviruses. Picornaviruses.

[17] Minor P.D., Ferguson M., Evans D.M.A., Almond J.W., Icenogle J.P. (1986). Antigenic Structure of Polioviruses of Serotypes 1, 2 and 3. J. Gen. Virol..

[18] Bannwarth L., Girerd-Chambaz Y., Arteni A., Guigner J.-M., Ronzon F., Manin C., Vénien-Bryan C. (2015). Mapping of the epitopes of poliovirus type 2 in complex with antibodies. Mol. Immunol..

[19] Patel V., Ferguson M., Minor P. (1993). Antigenic Sites on Type 2 Poliovirus. Virology.

[20] Dunn G., Klapsa D., Wilton T., Stone L., Minor P.D., Martin J. (2015). Twenty-Eight Years of Poliovirus Replication in an Immunodeficient Individual: Impact on the Global Polio Eradication Initiative. PLoS Pathog..

[21] Sidhu S.S. (2001). Engineering M13 for phage display. Biomol. Eng..

[22] Bever C.S., Dong J.-X., Vasylieva N., Barnych B., Cui Y., Xu Z.-L., Hammock B.D., Gee S.J. (2016). VHH antibodies: Emerging reagents for the analysis of environmental chemicals. Anal. Bioanal. Chem..

[23] Mitchell L.S., Colwell L.J. (2018). Comparative analysis of nanobody sequence and structure data. Proteins Struct. Funct. Bioinform..

[24] Strauss M., Schotte L., Thys B., Filman D.J., Hogle J.M. (2016). Five of Five VHHs Neutralizing Poliovirus Bind the Receptor-Binding Site. J. Virol..

[25] Strauss M., Schotte L., Karunatilaka K.S., Filman D.J., Hogle J.M. (2017). Cryo-electron Microscopy Structures of Expanded Poliovirus with VHHs Sample the Conformational Repertoire of the Expanded State. J. Virol..

[26] Ermakova M., Ivanov S., Shmeleva O., Borisenko N., Zyrina A., Chumakov S., Levin I., Yakupova R., Merkulova M., Shishova A. (2026). A Panel of VHH Antibodies Against Sabin Type 1 Poliovirus D-Antigen Reveals Strain-Specific and Cross-Serotype Reactivity. Viruses.

[27] Piniaeva A.N., Kovpak A.A., Ivin Y.Y., Sandzhieva S.H., Shishova A.A., Tcelykh I.O., Vasilenko V.E., Kaa K.V., Mazhed Z.H., Khapchaev Y.K. (2022). Application of Ion Exchange Chromatography in the Development of Technology to Obtain Inactivated Poliovirus Vaccine. Epidemiol. Vaccinal Prev..

[28] Zyrina A., Shishova A., Tcelykh I., Levin I., Shmeleva O., Borisenko N., Ermakova M., Ivanov S., Kovpak A., Vasilenko V. (2025). Generation of Polyclonal Antibodies Against Sabin Poliovirus D- and H-Antigens and Their Application in ELISA. Vaccines.

[29] World Health Organization (2004). Polio Laboratory Manual.

[30] Fustinoni C., Liu X., Chen Z., Cawood R., Valenti W., Patrício M.I., Su W. (2025). Self-silencing adenovirus enables precise infectious titration of recombinant adeno-associated viral vectors. Mol. Ther. Methods Clin. Dev..

[31] Baselga M., Sánchez-Prieto J., Medina Pérez V.M., Schuhmacher A.J. (2025). Species-Dependent Structural Variations in Single-Domain Antibodies. Antibodies.

[32] Sharma S., Mittal B., Vatsa R., Singh B. (2011). Gel clot bacterial endotoxin test of FDG: Indian scenario. Indian J. Nucl. Med..

[33] Martiín J., Samoilovich E., Dunn G., Lackenby A., Feldman E., Heath A., Svirchevskaya E., Cooper G., Yermalovich M., Minor P.D. (2002). Isolation of an Intertypic Poliovirus Capsid Recombinant from a Child with Vaccine-Associated Paralytic Poliomyelitis. J. Virol..

[34] Crainic R., Couillin P., Blondel B., Cabau N., Boué A., Horodniceanu F. (1983). Natural variation of poliovirus neutralization epitopes. Infect. Immun..

[35] Baca-Estrada M., Griffiths E. (2006). Regulation and standardization of IPV and IPV combination vaccines. Biologicals.

[36] Goswami P., Saini D., Sinha S. (2009). Phage Displayed scFv: pIII Scaffold May Fine Tune Binding Specificity. Hybridoma.

[37] Kaku Y., Noguchi A., Okutani A., Inoue S., Tanabayashi K., Yamamoto Y., Hotta A., Suzuki M., Sugiura N., Yamada A. (2012). Altered specificity of single-chain antibody fragments bound to pandemic H1N1-2009 influenza virus after conversion of the phage-bound to the soluble form. BMC Res. Notes.

[38] Giersberg M., Floss D.M., Kipriyanov S., Conrad U., Scheller J. (2009). Covalent dimerization of camelidae anti-human TNF-alpha single domain antibodies by the constant kappa light chain domain improves neutralizing activity. Biotechnol. Bioeng..

[39] Yao H., Song Y., Huang Q., Zhu M., Liang J., Zhang Z., Zhang X., Dong D., Shi D., Wu Z. (2026). Cryo-ET of IgG bivalent binding on SARS-CoV-2 provides structural basis for antibody avidity. Nat. Commun..

