# Supplementary material for: Generation and Characterization of Single-Domain Antibodies Against the D-Antigen of Sabin Poliovirus Types 2 and 3
Source: Viruses. 2026 Oct 16;18(10):1147. doi: 10.3390/v18101147 (PMC13709865; doi:10.3390/v18101147)
Supplement: Supplementary file 1 [file viruses-18-01147-s001.zip › viruses-4505754-supplementary.pdf]

Article

Generation and Characterization of Single-Domain Antibodies Against the D-Antigen of Sabin Poliovirus Types 2 and 3

Maya Ermakova [1,*,†], Sergey Ivanov [1,†], Olga Shmeleva [1], Nadezhda Borisenko [1], Anna Zyrina [1], Igor Levin [1], Stepan Chumakov [2], Anna Shishova [1,3,*], Yury Ivin [1], Anastasia Piniaeva [1], Alexandra Siniugina [1] and Aydar Ishmukhametov [1,3]

1 Chumakov Federal Scientific Center for Research and Development of Immune-and-Biological Products, Russian Academy of Sciences (Institute of Poliomyelitis), 108819 Moscow, Russia; ivanov_sv@chumakovs.su (S.I.)
2 Shemyakin-Ovchinnikov Institute of Bioorganic Chemistry, Russian Academy of Sciences (IBCh RAS), 117997 Moscow, Russia
3 Institute for Translational Medicine and Biotechnology, First Moscow State Medical University (Sechenov University), 119991 Moscow, Russia
* Correspondence: ermakova_mj@chumakovs.su (M.E.); a_shishova@list.ru (A.S.)
† These authors contributed equally to this work.

Supplementary Materials

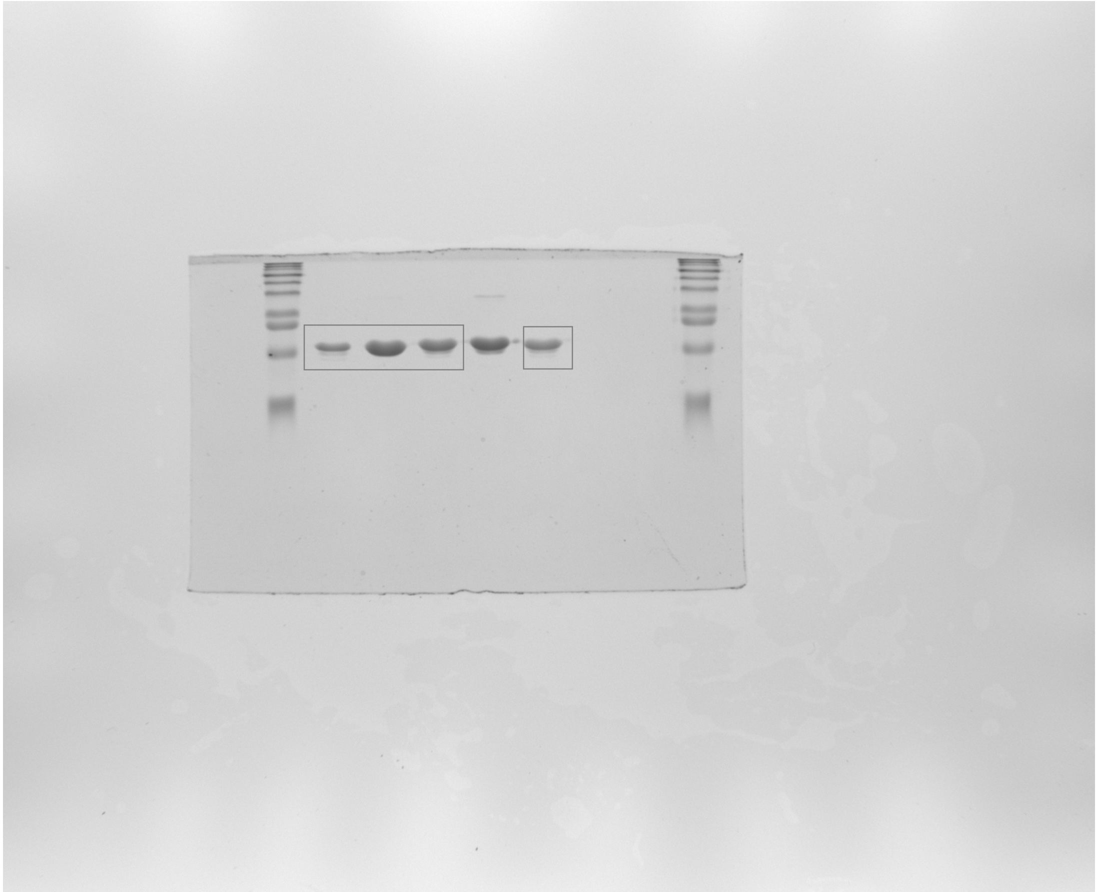

Supplementary Figure S1. SDS-PAGE gel showing purification of recombinant VHHs: S2-A1, S2-C5, S2-D5, S2-G9. The band corresponding to the purified VHH antibody (~20 kDa) used for Figure 3 is indicated by the box.

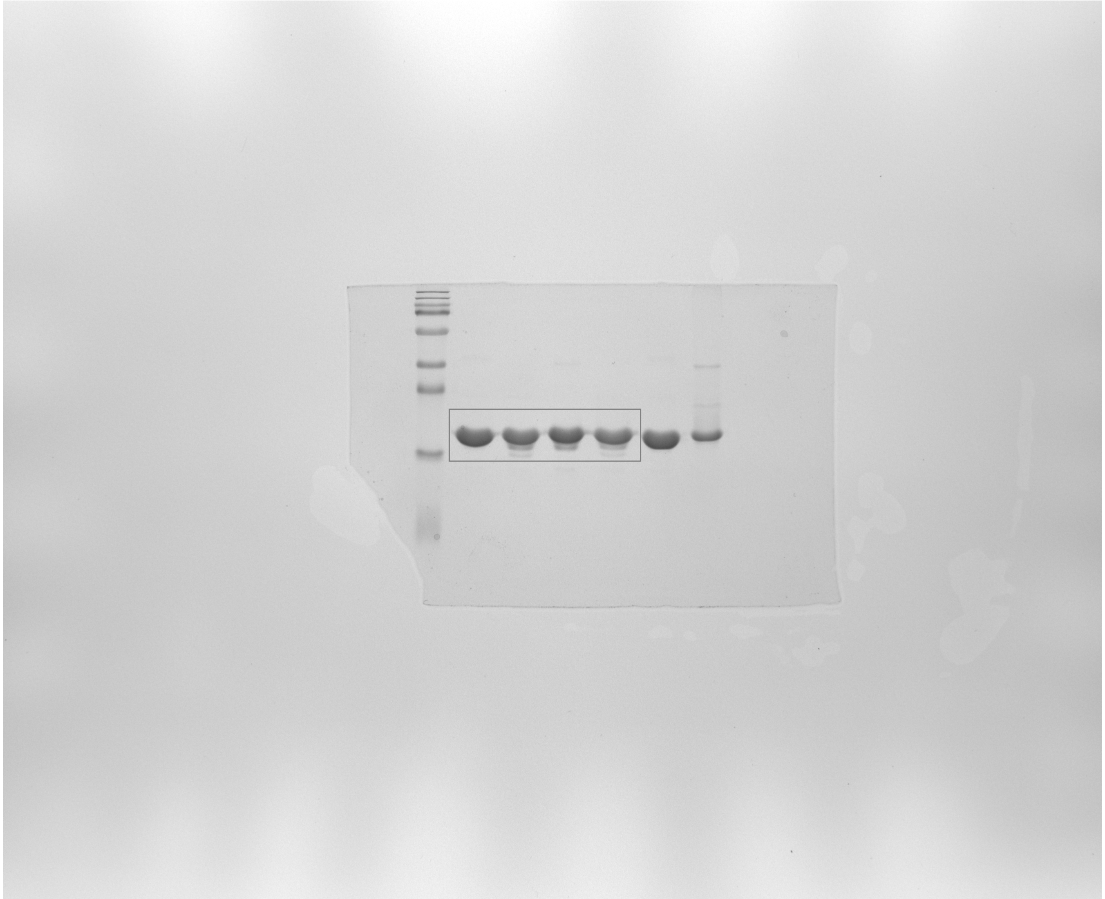

Supplementary Figure S2. SDS-PAGE gel showing purification of recombinant VHHs: S3-A2, S3-B5, S3-H1, S3-G1. The bands corresponding to the purified VHH antibodies (~20 kDa) used for Figure 3 are indicated by boxes.

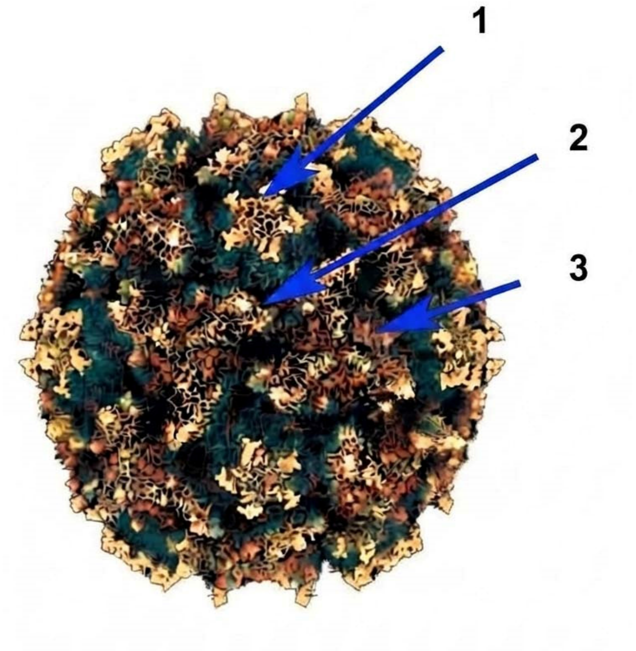

Supplementary Figure S3. Antigenic structure of poliovirus. Antigenic site 1 (1) is located in VP1 (residues 89-100) and is immunodominant for serotypes 2 and 3, whereas it is immunorecessive for serotype 1. **Site 2 (2)** is a complex, discontinuous epitope combining residues 220-222 of VP1 (subsite 2a) with residues 164-172 and 270 of VP2 (subsite 2b). Both subsites are active in serotypes 1 and 2, while in serotype 3 only subsite 2b is distinctly detected. **Site 3 (3)** is also complex and comprises subsite 3a in VP1 (residues 286-290) and subsite 3b in VP3 (residues 58-59 and others). Both subsites 3a and 3b are present in serotypes 2 and 3, whereas only subsite 3b has been found in serotype 1.

		2A1											
		SI	SII	SIII	SI (H)	SII (H)	SIII (H)	Mahoney	MEF-1	Saukett	Mah (H)	MEF (H)	Sauk (H)
Du/ml		1	2	3	4	5	6	7	8	9	7	8	9
50	A	0,0566	1,0947	0,2627	0,0712	1,8921	0,4253	0,0693	0,0590	0,0623	0,0520	0,0546	0,0515
16,667	B	0,0806	0,2886	0,2852	0,0589	1,1184	0,1927	0,0662	0,0546	0,0598	0,0507	0,0507	0,0508
5,556	C	0,0745	0,1275	0,2662	0,0647	0,6215	0,1189	0,0605	0,0531	0,0537	0,0507	0,0521	0,0510
1,852	D	0,0790	0,0716	0,2626	0,0583	0,1782	0,0558	0,0583	0,0516	0,0531	0,0507	0,0504	0,0509
0,617	E	0,0737	0,0560	0,2631	0,0535	0,1023	0,0543	0,0638	0,0521	0,0526	0,0509	0,0524	0,0513
0,206	F	0,0720	0,0504	0,2626	0,0556	0,0628	0,0496	0,0619	0,0529	0,0535	0,0514	0,0513	0,0506
0,069	G	0,0756	0,0496	0,2631	0,0538	0,0608	0,0505	0,0549	0,0525	0,0505	0,0511	0,0511	0,0517
0	H	0,0501	0,0497	0,0523	0,0441	0,0498	0,0449	0,0505	0,0500	0,0503	0,0506	0,0504	0,0505

		2C5											
		SI	SII	SIII	SI (H)	SII (H)	SIII (H)	Mahoney	MEF-1	Saukett	Mah (H)	MEF (H)	Sauk (H)
Du/ml		1	2	3	4	5	6	7	8	9	7	8	9
50	A	0,0667	3,4126	0,0804	0,0795	0,0845	0,0808	0,0576	1,0102	0,0508	0,0520	0,0546	0,0514
16,667	B	0,0531	2,5545	0,0755	0,0786	0,0818	0,0762	0,0516	0,7295	0,0519	0,0507	0,0507	0,0516
5,556	C	0,0504	1,8349	0,0761	0,0761	0,0751	0,0783	0,0518	0,3941	0,0552	0,0507	0,0521	0,0512
1,852	D	0,0505	0,7699	0,0747	0,0758	0,0769	0,0791	0,0540	0,1469	0,0513	0,0507	0,0504	0,0512
0,617	E	0,0512	0,2972	0,0749	0,0726	0,0731	0,0803	0,0510	0,0833	0,0533	0,0509	0,0524	0,0516
0,206	F	0,0498	0,1910	0,0737	0,0731	0,0783	0,0759	0,0508	0,0623	0,0635	0,0514	0,0513	0,0523
0,069	G	0,0520	0,0727	0,0766	0,0708	0,0709	0,0732	0,0543	0,0551	0,0581	0,0511	0,0511	0,0507
0	H	0,0507	0,0507	0,0507	0,0504	0,0510	0,0517	0,0506	0,0510	0,0538	0,0506	0,0504	0,0511

		2D5											
		SI	SII	SIII	SI (H)	SII (H)	SIII (H)	Mahoney	MEF-1	Saukett	Mah (H)	MEF (H)	Sauk (H)
Du/ml		1	2	3	4	5	6	7	8	9	7	8	9
50	A	0,0890	2,6623	0,2245	0,0997	0,7658	0,3938	0,0581	0,7361	0,0556	0,0542	0,1410	0,0532
16,667	B	0,0581	1,8254	0,1163	0,0780	0,4712	0,2581	0,0538	0,4519	0,0542	0,0538	0,0710	0,0526
5,556	C	0,0507	1,2911	0,0935	0,0564	0,1954	0,2204	0,0517	0,2319	0,0523	0,0501	0,0576	0,0524
1,852	D	0,0505	0,5927	0,0880	0,0526	0,0965	0,2036	0,0513	0,1173	0,0531	0,0501	0,0517	0,0565
0,617	E	0,0495	0,2186	0,0883	0,0492	0,0578	0,1908	0,0533	0,0735	0,0529	0,0503	0,0498	0,0551
0,206	F	0,0494	0,0716	0,0865	0,0515	0,0521	0,1785	0,0516	0,0598	0,0532	0,0494	0,0506	0,0670
0,069	G	0,0503	0,0673	0,0853	0,0528	0,0504	0,1702	0,0528	0,0552	0,0540	0,0503	0,0511	0,0809
0	H	0,0504	0,0523	0,0550	0,0493	0,0495	0,0513	0,0588	0,0532	0,0533	0,0495	0,0512	0,0851

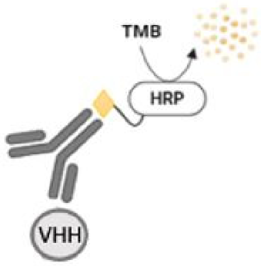

Supplementary Figure S4. Raw ELISA data demonstrating the absence of cross-reactivity between capture VHH antibodies and detection polyclonal antibodies. The assay layout replicates the experimental setup used for specificity assessment (Figures 4-7), including all components of the sandwich ELISA: immobilized VHH antibodies, serotype-specific biotinylated rabbit polyclonal antibodies, and streptavidin-HRP conjugate. The bottom row of the table (highlighted in orange) represents the background control, in which the antigen was omitted from the corresponding wells. Any signal detected in the absence of antigen would indicate non-specific binding of the detection polyclonal antibodies to the capture VHHs or to the plate surface. As shown, the background signals in the red-highlighted wells are uniformly low and indistinguishable from the blank values, confirming that the detection antibodies do not interact with the immobilized VHHs and do not produce non-specific background under the assay conditions.

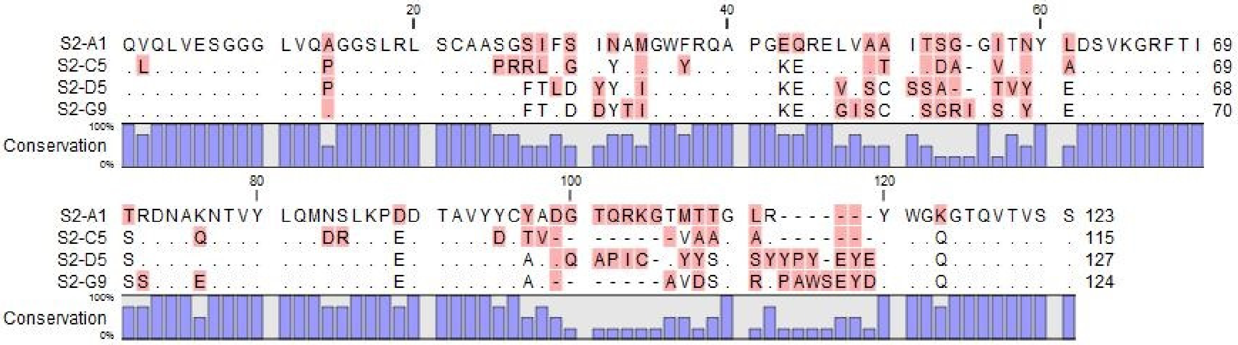

Supplementary Figure S5. Multiple sequence alignment of the amino acid sequences of nanobody clones selected against poliovirus Sabin type 2, generated with CLC Sequence Viewer (QIAGEN).

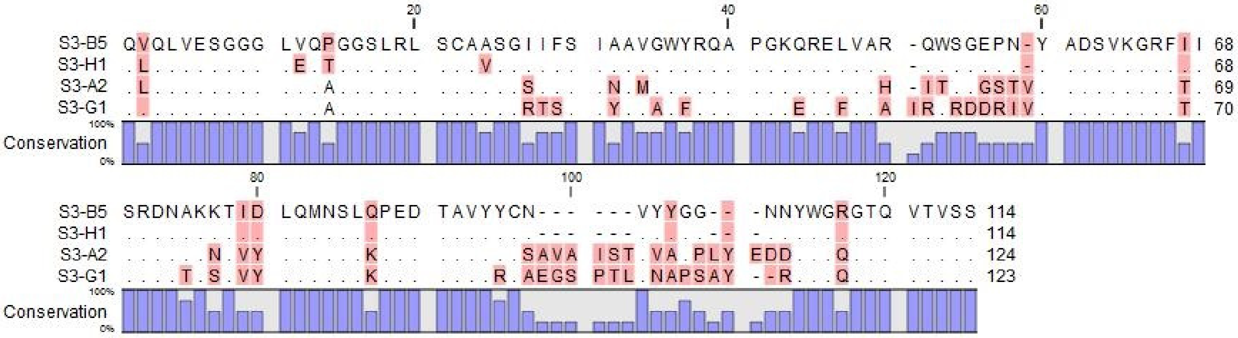

Supplementary Figure S6. Multiple sequence alignment of the amino acid sequences of nanobody clones selected against poliovirus Sabin type 3, generated with CLC Sequence Viewer (QIAGEN).
